# Double-Needle Meniscal Suture Technique: Technical Description and Clinical Application in Dogs

**DOI:** 10.3390/ani14182717

**Published:** 2024-09-19

**Authors:** Gian Luca Rovesti, Beatrice Böhme

**Affiliations:** 1 Clinica Veterinaria M.E. Miller, Via della Costituzione, 10, 42025 Cavriago, Italy; gl.rovesti@clinicamiller.it; 2 Kleintierzentrum Hirschberg, Landstr. 15, 69493 Hirschberg, Germany

**Keywords:** arthroscopy, stifle, meniscal surgery, meniscal suture, dog

## Abstract

**Simple Summary:**

Cranial cruciate ligament (CCL) disease often involves damage to the caudal horn of the medial meniscus, resulting in pain and the progression of osteoarthritis. Current treatments, primarily partial caudal meniscectomy, have shown disappointing long-term outcomes as osteoarthritis progresses. Consequently, restoring and preserving meniscal tissue is becoming increasingly important for maintaining the integrity and functionality of the meniscus whenever possible. Although meniscal suture techniques have been documented in canine cases, their clinical application remains limited. This study aims to assess the practicability of a minimally invasive double-needle technique (DNT) designed to facilitate meniscal sutures during arthroscopy with the use of joint distraction. The technique was applied in ten canine stifles undergoing arthroscopy for CCL disease with confirmed medial meniscal lesions of the caudal horn appropriate for suturing. The described DNT proved to be a viable method for suturing the caudal horn of the medial meniscus. It can be effectively applied in canine stifles, provided there is adequate visualization during the procedure.

**Abstract:**

Current treatments for medial meniscus lesions in association with CCL ruptures have shown disappointing long-term outcomes. Meniscal suturing may improve the outcome, but their clinical application remains limited. This study aims to assess the practicability of a minimally invasive DNT to facilitate meniscal suturing in dogs. Ten stifles of eight client-owned dogs with arthroscopically confirmed CCL disease and medial meniscal tears in the abaxial third of the meniscus were included. The described suture technique was applied under joint distraction using the Titan joint distractor. Surgical stabilization of all stifles was then accomplished via an X-Porous TTA procedure. The DNT allowed for the precise placement of meniscal sutures. Minor intraoperative complications included reduced arthroscopic visibility (*n* = 2) and suture breakage during its passage through the meniscus (*n* = 3). No complications related to the meniscal sutures were noted throughout the six-month follow-up period. The described DNT proved to be a viable and effective method for suturing lesions of the caudal horn of the medial meniscus, provided there is adequate visualization during the procedure. Appropriate stifle stabilization postoperatively is mandatory for protecting the suture and avoiding concomitant meniscal lesions due to joint instability.

## 1. Introduction

Cranial cruciate ligament disease is a common reason for hind limb lameness in dogs and is frequently associated with medial meniscal lesions at the time of surgery (ranging from 20 to 70% of cases [1,2,3,4,5]). Conversely, lateral meniscal lesions are less frequent, occurring in 2% of dogs with partial or complete cranial cruciate ligament ruptures [1,2,3,6]. The menisci play a crucial role in the stifle joint ensuring joint congruency [2], aiding in load distribution, contributing to articular cartilage lubrication, and preventing synovial entrapment during weight bearing [7,8]. The medial meniscus is attached to the tibia, joint capsule, and medial collateral ligament, whereas the lateral meniscus is more mobile. Damage of the medial meniscus often occurs in association with complete cranial cruciate ligament tears causing instability in the stifle joint. This occurs when the medial meniscus is compressed between the femoral condyle and tibial plateau during cranial displacement of the tibia, leading to shear forces exacerbated by internal rotation, and frequently leading to meniscal damage [7]. Various diagnostic methods such as ultrasonography, arthrography, magnetic resonance imaging (MRI), and arthrotomy have been utilized to examine the menisci, with arthroscopy considered the gold standard for stifle joint assessment [6,7,9,10]. The most commonly reported lesions are fibrillation of the surface and compression injuries, longitudinal tears and bucket handle tears, radial or transverse tears, horizontal, oblique, or flap tears, complex macerated tears, and a folded caudal horn [2,7,11,12,13]. Current treatments depend on the type of injury and range from partial caudal meniscectomy and caudal hemimeniscectomy to total meniscectomy, aimed at removing damaged tissue and alleviating pain [1,11,14,15,16]. Partial meniscectomy, favored for less degenerative changes than meniscectomy, has become the most common treatment [3,7,17,18]. However, resection still compromises meniscal function and results in stress concentration, possibly predisposing to osteoarthritis [4,19]. Studies in humans have shown that meniscal suturing can lead to less osteoarthritic progression, reduced pain, and improved long-term function compared to meniscectomy [20]. Some studies emphasizing the importance of sparing the meniscus have also been performed in dogs: one study reveals that an intact lateral meniscus transmits 29% of the load. In the case of lateral partial meniscectomy with three-quarters of the lateral meniscus remaining, the load transmission increases to 45%. After total meniscectomy, the load transmission increases to 313%, a 7-fold increase in load passing through the remaining structures [7]. Other studies have found a 2.5-fold increase in the area of peak pressure on the tibial plateau after medial caudal pole meniscectomy. In cases of cranial cruciate deficient stifles treated by tibial plateau leveling osteotomy (TPLO), a 1.7-fold increase was still noted after medial caudal pole meniscectomy [4].

**Figure 1:**
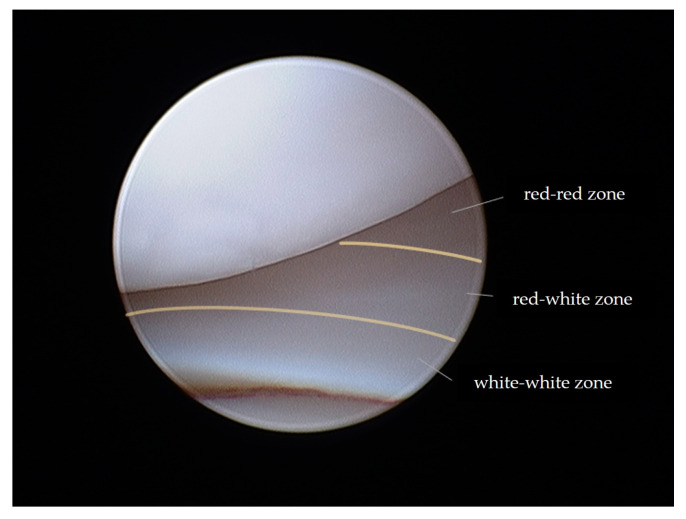
Meniscus. The red zone or red–red zone is the vascularized peripheral third of the meniscus, the red–white zone in the middle part is less vascularized, and the inner white–white zone is the non-vascularized portion of the meniscus.

Given the potential adverse effects of meniscectomy, efforts have been made to develop meniscal repair techniques that preserve meniscal tissue and promote healing [3,21,22]. The meniscus’s limited vascularization restricts healing to the vascularized red–red zone. It is divided into three zones depending on the vascularization pattern. The inner avascular white–white zone with low to no healing potential, the red–red zone on the outer rim with vascularization and good healing potential, and the red–white zone in between (Figure 1) with lower vascularization and lower healing potential [16,23,24].

Concerning suture knot types, Thieman et al. [25] evaluated three different meniscal repair techniques for the restoration of femorotibial contact mechanics in a cadaveric dog stifle model with bucket handle tears of the medial meniscus. Sutured menisci restored by vertical, horizontal, and cruciate sutures were compared. No difference was detected in restoring the contact area, the mean contact pressure, and the peak contact pressure between the suture knot types used [25]. The restoration of mean contact pressure and peak contact pressure after suture repair was improved for repaired menisci independent of the suture knot types when compared to partial meniscectomy and closer to healthy controls than after partial meniscectomy. All repair techniques restored normal contact mechanisms of the medial compartment. In contrast, partial meniscectomy caused a 35% decrease in the contact area, a 57% increase in the mean contact pressure, and a 55% increase in peak contact pressure compared to the intact meniscus [25].

In human medicine, arthroscopic meniscal repair techniques are the gold standard for suturing meniscal injuries [20,26] and various techniques have been documented [20,26,27,28,29,30]. The outside-in meniscal repair technique for treating anterior and mid-body tears was already introduced in 1985 by Warren et al. [30]. The needles are passed from outside the joint capsule through the two fragments of the meniscus, and both extremities of the suture are retrieved at the outside of the joint capsule to tie the knot extra-articularly [20,26]. The inside-out technique, applicable for mid-body and posterior tears, entails threading sutures from inside the joint through both segments of the tear before passing them through the capsule. These sutures are then retrieved outside the joint and secured over the capsule using a minor open approach [20]. Additionally, fully arthroscopic all-inside repair techniques have been detailed, employing various suture devices [20,31]. These devices facilitate the introduction and tightening of knots entirely from within the joint [20], eliminating the need for an open approach, thereby saving time and minimizing pain [20].

A major concern for implementing arthroscopic or arthroscopic-assisted meniscal repair techniques in dogs is the limited joint space. Suture knots are therefore preferably placed as capsular-side knots outside of the joint [12]. Techniques such as shaver, electrocoagulation, and joint distraction have been detailed to enhance visualization and working space in the canine stifle, facilitating complex procedures like meniscal suturing [12,13,22,32,33,34].

Moses et al. described an open, modified ”inside-out” technique in dogs, used for caudal peripheral detachment and longitudinal tears of the medial meniscus [35]. Suture strands were tightened by a knot on the outside of the joint capsule and a second mattress suture was placed accordingly as required [35]. The performance and description of arthroscopically performed meniscal sutures in dogs are still rare [12,22,36,37].

Our study aimed to evaluate the feasibility of an arthroscopic-assisted double-needle technique for meniscal sutures in the caudal horn of the medial meniscus in dogs under appropriate joint distraction.

## 2. Materials and Methods

### 2.1. Study Population and Inclusion Criteria

Client-owned dogs (*n* = 8) presented to the Clinica M. E. Miller in Cavriago, Italy, for hind limb lameness assessment were included in the study when meeting the inclusion criteria of cranial cruciate ligament rupture and medial meniscal lesions in the abaxial third of the medial meniscus amenable to meniscal repair by meniscal suturing using the double-needle technique described (*n* = 10).

The breeds were Cane Corso (*n* = 2), Labrador (*n* = 1), Dobermann (*n* = 1), German Shorthair (*n* = 1), Boxer (*n* = 1), Maremmano-abruzzese (*n* = 1), and mixed breed (*n* = 1) with an average age of 5.28 years (range, from 1.8 to 11.5 years). Two of the dogs underwent treatment for both limbs at different times. The mean body weight was 36 kg ± 7.1 kg (range 24 to 48 kg). Three male and five female dogs with six left and four right stifles were included in the study. Clinical and radiographic evaluations were performed alongside preoperative blood tests. A complete cranial cruciate ligament tear and a concurrent lesion of the caudal horn of the medial meniscus suitable for meniscal suture repair were confirmed via arthroscopic examination. The meniscal lesion was considered appropriate for meniscal suture treatment when it was located in the abaxial third of the meniscus (red–red zone, Figure 1), with enough healthy tissue present to ensure sufficient holding force for the sutures.

General anesthesia was initiated intravenously with Midazolam and Propofol and maintained with isoflurane in oxygen. Perioperative pain medication included subcutaneous meloxicam (Meloxicam Injection^®^ 20 mg/mL, Dechra, Putney Inc., Portland, OR, USA) and intravenous buprenorphine at the time of induction (buprenorphine 0.01 mg/kg, Buprenodale^®^, Dechra limited, Stoke-on-Trent, UK). Perioperative antibiotic prophylaxis was provided with a single intravenous dose of Cefazoline (20 mg/kg, Cefamezin^®^, Pfizer Inc., New York, NY, USA) at the time of induction up to 2022. After that date, no antibiotic prophylaxis was used if the procedure was uneventful and completed within two hours of surgery.

### 2.2. Arthroscopic Procedure

Arthroscopy of the stifle joint was conducted in dorsal recumbency with the dog positioned in a foam cushion, the unaffected hind limb secured in an abducted position, and the affected limb freely hanging for manipulation. Preliminary evaluation of the joint was initially performed arthroscopically without distraction. After detection of the meniscal lesion or if other abnormalities required further evaluation, the joint distractor was applied to the limb. Joint distraction was achieved using the Titan distractor device (Titan distractor, Ad Maiora, Cavriago, Italy), facilitating the insertion of arthroscopic instruments. The distractor was applied as described previously [12]. A 2.4 mm 30° fore-oblique arthroscope and a palpation hook were, respectively, inserted through cranio-medial and craniolateral portals.

The level of distraction was meticulously adjusted during the procedure to ensure adequate visualization of the meniscus. This enabled precise diagnosis and the execution of necessary meniscal suture maneuvers tailored to the patient’s needs.

### 2.3. Meniscal Sutures

**Figure 2:**
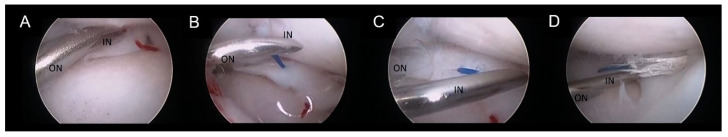
Double-needle suture technique. Intraoperative picture of different steps of meniscal suture repair using the double-needle technique in a right stifle joint: (**A**) The double-needle construct is placed in position, and the spinal needle (IN) is passed through the meniscus, while the 16G needle (ON) is holding the meniscus in position. (**B**) The spinal needle is retrieved within the joint holding the suture. (**C**) The spinal needle drives the strand of the suture in a different area of the meniscus over the lesion. (**D**) The suture loop is passed through the meniscus more abaxially and outside the joint to the caudo-medial area of the stifle where the needle is retrieved.

**Figure 3:**
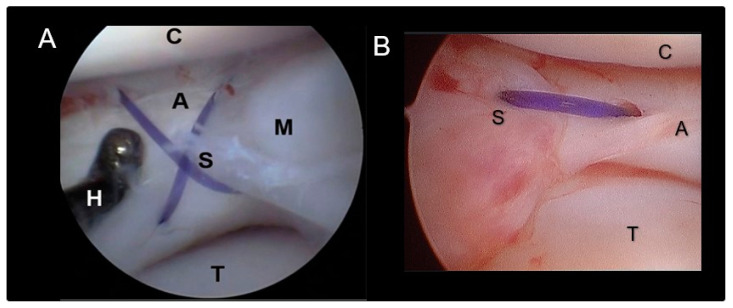
Suture knot types. (**A**) Cross stitch meniscal suture. (**B**) Horizontal stitch meniscal suture; A + M. meniscus, S—suture/stitches, C—cartilage of the medial femoral condyle, T—cartilage of the tibial plateau.

Suture application for meniscal tears was performed in meniscal lesions located in the abaxial third of the meniscus (red–red zone, as illustrated in Figure 1) when an adequate amount of healthy tissue was present to ensure a robust suture hold. The suturing technique employed was the double-needle method, an evolution of the single-needle technique already described [12]. A 22-G spinal needle (inner needle, IN) was passed through the lumen of a 16-G standard needle (outer needle, ON) (Figure 2). The assembly was introduced through the cranio-lateral portal and navigated in a caudo-medial direction until contact with the meniscal surface was reached. Subsequently, the spinal needle was advanced through the meniscus plan to the lesion and across the lesion site (Figure 2A). It was then pushed through the joint capsule, emerging in the caudo-medial aspect of the stifle joint, where the needle tip was palpated and exposed by a stab incision. Soft tissue dissection was meticulously performed to reveal the needle tip. The needle trocar was removed. The suture material was then inserted into the inner needle’s tip and advanced until caught at the needle cone, secured by manual grasp. Retracting the inner needle tip back into the joint rendered the suture strand visible within the joint (Figure 2B). While the outer needle maintained meniscal stability and kept a working distance between the meniscus and the scope, a suture loop was created. The inner needle’s bevel was rotated. The spinal needle was once more propelled through the meniscus abaxial to the initial insertion (Figure 2C,D), pushing the suture through the meniscus. Vigilance was exercised to prevent suture damage by the needle’s tip. In case of suture damage, the procedure was repeated. Upon the needle tip’s re-emergence at the joint capsule, the suture loop was retrieved from the needle tip, and the extremity was pulled out, leaving the two extremities of the suture exiting from the wound. Following needle removal, the two suture ends were carefully tightened without tension by a square knot and 4–5 secure knots, ensuring the meniscal segments were closely apposed without gap formation or bulging. Sutures were applied in cross, horizontal, or vertical configurations (Figure 3). The process was repeated as many times as needed to meet clinical requirements (Figure 2 and Figure 3) and achieve a secure hold by the sutures. The choice of suture knot type and material was tailored to each patient, with 2–4 crossed and horizontal or vertical mattress sutures executed using polypropylene (Prolene^®^ USP 3–0, Ethicon, Raritan, NJ, USA) and/or polydioxanone (PDS II^®^, USP 3–0, Ethicon, Raritan, NJ, USA) (Table 1). Any complication encountered was recorded.

### 2.4. Surgical Stabilization of the Stifle Joint

**Figure 4:**
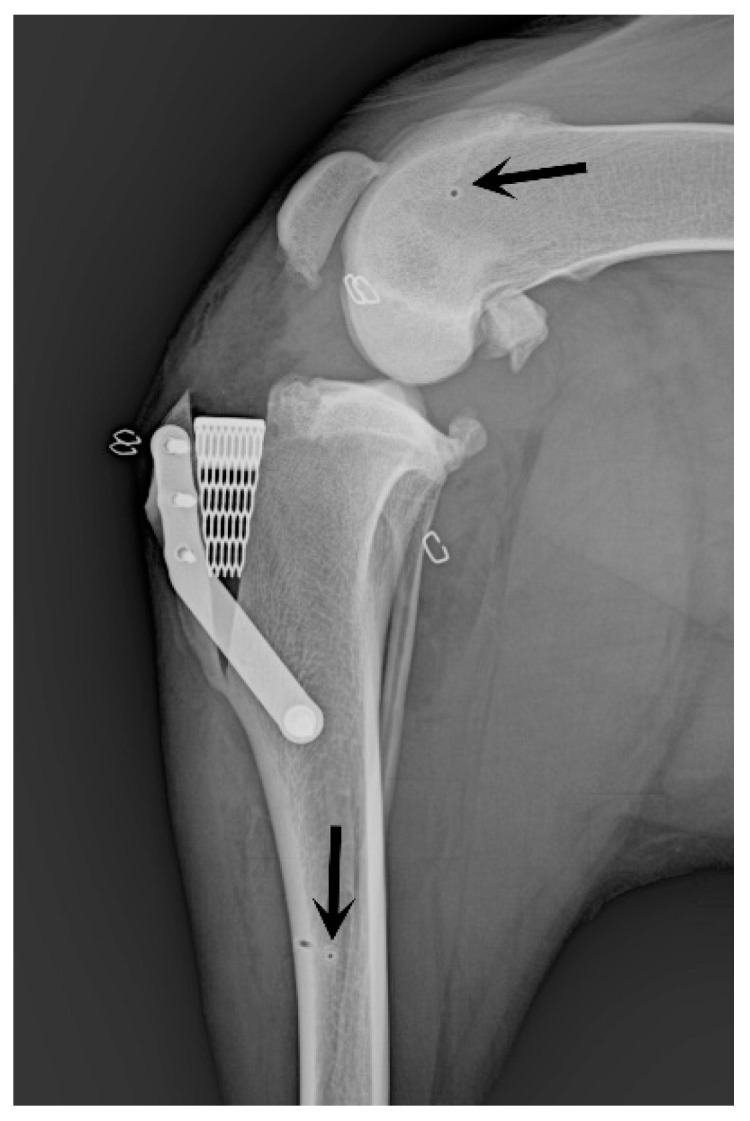
Postoperative X-Porous TTA mediolateral radiographic projection. Note the small holes due to the K wires of the traction stirrups connected to the Titan distractor.

The stifle joint underwent surgical stabilization to treat cranial cruciate ligament injuries, employing the X-Porous tibial tuberosity advancement (X-Porous TTA) procedure [38] (implants: Ad Maiora, Cavriago, Italy). Post-surgical assessments included radiographic evaluations for all cases (Figure 4).

### 2.5. Postoperative Management

Dogs were discharged as soon as they recovered from general anesthesia and vital parameters were stable. The postoperative analgesic regimen included meloxicam (Meloxoral^®^, 0.1 mg/kg once daily, PO, A.T.I. s.r.l. Ozzano Emilia, Italy) administered over two weeks and tramadol (Altadol^®^, Formevet s.r.l., Milano, Italy; 3 mg/kg) dispensed for five days. Cephalexin (ICF Vet^®^, I.C.F. Industria Chimica Fine s.r.l. Pignano, Italy; 20 mg/kg twice daily) was prescribed as antibiotic coverage for one week up to 2022; afterwards, no antibiotic was prescribed if the procedure was considered uneventful. All dogs were managed without bandages and were subjected to restricted and controlled activity. They were limited to leash walks of specified duration, three to four times daily over an eight-week period. Cryotherapy (application of cold packs) and massage, especially in cases with postoperative edema of the ipsilateral tarsus, were applied postoperatively.

### 2.6. Postsurgical Follow Up

Routine clinical and radiographic evaluations were scheduled at 10 and 45 days postoperatively, with subsequent assessments based on the surgeon’s judgment until bone healing. The follow-up period was on average 6 months. A second-look arthroscopy was not performed in any case.

## 3. Results

Eight client-owned dogs presented for hind limb lameness met the inclusion criteria of cranial cruciate ligament rupture and medial meniscal lesions in the abaxial third of the medial meniscus amenable to meniscal repair by meniscal suturing using the double-needle technique described (*n* = 10).

The breeds included were Cane Corso (*n* = 2), Labrador (*n* = 1), Dobermann (*n* = 1), German Shorthair (*n* = 1), Boxer (*n* = 1), Maremmano-abruzzese (*n* = 1), and mixed breed (*n* = 1) with an average age of 5.28 years (range, from 1.8 to 11.5 years). Two of the dogs underwent treatment for both limbs at different times. The mean body weight was 36 kg ± 7.1 kg (range 24 to 48 kg). Three male and five female, six left and four right stifles were included in the study. Preoperative blood tests showed no abnormalities. Presurgical radiographic evaluation revealed joint effusion of the stifle in all dogs. The arthroscopic assessment was performed in all dogs without distraction for preliminary evaluation. When the meniscal lesion was detected or some abnormality required further evaluation, the distractor was applied to the limb. Sufficient distraction allowed for the evaluation of both menisci in all cases with slight difficulties in visualization in two cases (Table 1). Findings confirmed a complete cranial cruciate ligament rupture in all dogs, and meniscal lesions were identified in the abaxial third of the caudal horn of the medial meniscus (Table 1, Figure 2). Utilizing the double-needle technique, meniscal suturing was successfully performed in all dogs with surgical times ranging from 42 to 83 min (average 64 min ± 22 min). The suturing was conducted by two stitches in four cases, three stitches in four cases, and four stitches in one case. Of the total number of twenty-seven stitches, twenty-one were vertical, two were horizontal, and four were crossed. The suture materials used were nonabsorbable polypropylene (sixteen stitches) and polydioxanone (eleven stitches) (Table 1).

Intraoperative complications included difficult visualization in two cases and suture cutting by the needle tip during the passage of the suture through the meniscus in three cases (11.11% of stitches, 5.55% of passages through the meniscus) (Table 1). No further complications were recorded when repeating the procedure.

Following meniscal repair, an X-Porous TTA (modified tibial tuberosity advancement) procedure was routinely performed in all dogs to restore stifle stability. The postoperative tibial compression test was negative in all dogs.

Postoperative clinical rechecks revealed no complications associated with the meniscal repair technique. Thirty percent of dogs developed edema at the level of the ipsilateral tarsus, which was treated with physical therapy (application of cold packs, massage) and resolved within 3–4 days without any sequel. One dog presented a tibial fracture one month after surgery for a reason unrelated to the study. The fracture was subsequently treated with plate osteosynthesis.

## 4. Discussion

Cranial cruciate ligament disease often involves damage to the caudal horn of the medial meniscus, resulting in pain and the progression of osteoarthritis. Given the potential adverse effects of meniscectomy and the attempt to avoid arthrotomy, arthroscopic meniscal repair is a standard procedure in humans [20,26,27,28,29,30] but still rarely described in dogs [12,22].

Moses et al. detailed a medial arthrotomy approach that involves lateral reflection of the patella to facilitate meniscal suturing [35]. The double-needle technique described in our study allowed us to avoid arthrotomy and required only a small stab incision at the caudomedial aspect of the joint for suture passage and tightening as mentioned above. Meniscal suturing was successfully performed in all cases. Despite concerns regarding the potential for greater iatrogenic injury described for arthroscopy [39], our experience suggests it is less invasive, offers superior visualization of the menisci, and simplifies the procedure compared to arthrotomy [12,35]. The Titan distractor provided adequate visualization in most cases [12,35]. In two stifles, visualization was reduced, but it was still sufficient for performing the procedure without iatrogenic damage. Cartilage damage was evaluated during the procedure, but no second-look arthroscopy was performed later on as it was denied by the owner. Cadaver studies may be an option to evaluate cartilage damage using specific stains to quantify the damage in a more objective way in the future [37].

The only intraoperative complication encountered was suture damage by the needle itself during passage through the meniscus. This complication was addressed by repeating the procedure and no further issues were reported.

Meniscal lesions were only considered appropriate for meniscal suture treatment when located in the abaxial third of the meniscus (red–red zone) when enough healthy tissue was present to ensure a robust suture hold, assuming that only tears in this zone would have the potential for healing. Because no re-evaluation was performed arthroscopically, no information about meniscal healing status or the presence of persistent non-union can be provided. Future research should investigate if healing occurs and if healing capacities in dogs are comparable to humans.

The type of suture stitches used does not seem to influence the restoration of meniscal tissue as shown by Thieman et al. [25]. The use of crossed, horizontal, or vertical sutures was therefore considered feasible and adapted to the specific lesion.

In early investigations, monofilament synthetic absorbable suture (polydioxanone. PDS II, size 1.5 metric) was described as a suture material for meniscal sutures [35], observing no clinical complication. No recommendations can be given for the choice of suture material in canine patients. The choice is not evidence-based and very indifferent, depending on technique and surgeon preference [22]. Further studies are required to determine which suture material allows healing without creating foreign body reactions within the joint and at the same time would provide enough strength until healing is finished. Additionally, the suture requires low elongation to prevent gap formation and a sufficient load to failure until healing has occurred [40]. The use of permanent sutures facilitates extended fixation periods necessary for the healing, maturation, and remodeling of the meniscus [40], despite the implication of leaving foreign material within the stifle joint. In early and still limited human studies, insufficient evidence supports that non-absorbable sutures in meniscus repair surgery enhance meniscal healing success rate compared to absorbable sutures [41]. Conversely, other hints exist that absorbable sutures promote meniscal healing [41]. In our study, we integrated the healing benefits with prolonged stability, utilizing absorbable sutures (polydioxanone) to foster healing alongside non-absorbable sutures (polypropylene) to ensure extended stability throughout the healing process. A constraint of our study is the challenge of definitively verifying holding capacity, suture integrity, and meniscal healing within a considerably long follow-up period.

Although follow-up arthroscopy could potentially help answer this question, the lack of clinical symptoms frequently results in owners’ hesitancy towards authorizing this supplementary procedure, given the requirements for anesthesia and the risk of inflicting additional surgical trauma on the dog. The small number of patients together with a short follow-up period of 6 months on average is part of the limitations of our study. None of the dogs showed recurrence of lameness or other clinical signs. In humans, 19% of meniscus repairs undergo revision, with failures often occurring beyond the second postoperative year. Anyway, it remains uncertain whether these failures are due to the meniscus repair itself or to additional adjacent tears [42].

Furthermore, it is important to emphasize the necessity of the stifle stabilization technique to mitigate the forces within a cruciate–ligament deficient stifle joint, which is crucial for enabling meniscal healing [35]. In our case–cohort, stability was achieved through a variant of the tibial tuberosity advancement technique, known as X-Porous TTA [38]. Other stabilization methods may be adequate if appropriate stability can be achieved [35]. The differences and choice of stabilization techniques available are beyond the focus of this study.

Meniscal sutures of the caudal horn of the medial meniscus were treatable by the arthroscopic assisted double-needle technique described. However, additional research is necessary to refine the suture technique, prevent subsequent damage, gain knowledge about the behavior of various suture materials in contact with synovial fluid, ascertain meniscal healing, and assess long-term clinical outcomes in a larger number of patients. Furthermore, mechanical testing and clinical studies are required to determine the most suitable suture material to prevent elongation and gap formation, enhance healing, and avoid intraarticular foreign body reactions in the long term. It is also important to understand how ambulation affects meniscal movements and the suture’s holding capacity.

## 5. Conclusions

In conclusion, the arthroscopic assisted double-needle technique described is a valuable and effective method for performing meniscal sutures in dogs’ caudal horns of the medial meniscus, given that appropriate visualization is achieved by adequate distraction. The outcome was good in all dogs without complications associated with the suture technique described. While further research is necessary to assess meniscal healing and long-term outcomes, our findings are encouraging and suggest a direction toward more conservative, tissue-preserving treatments that aim to maintain structural and functional meniscal integrity whenever possible.

## Figures and Tables

**Table 1:** Clinical data, suture material and types used, time to complete meniscal sutures, complications, and concomitant surgical procedures.

*N*.	Breed	Sex M/F	Body Weight (kg)	Age at Surgery	Stifle	Concomitant Disease	Meniscal Lesion	Suture	Suture Material	Time for Meniscal Suture (min)	Suture Complication	Concomitant Surgical Procedure
1	Maremmano-abruzzese	F	42	5 y 5 m	Left	CCL rupture	Caudal horn medial meniscus	3 stitches: 2 vertical, 1 horizontal	1 polypropylene 2 PDS	48	Breakage of one PDS suture during insertion through meniscus	X-Porous TTA
2	Shorthair	F	24	11 y 6 m	Right	CCL rupture	Caudal horn medial meniscus	2 stiches: 2 vertical	2 polypropylene	65	Difficult visualization	X-Porous TTA
3	Cane Corso	F	40	1 a 8 m	Right	CCL rupture	Caudal horn medial meniscus	4 stitches: 2 crossed, 2 vertical	2 polypropylene 2 PDS	78	None	X-Porous TTA
4	Cane Corso	F	40	2 y	Left	CCL rupture Tibia fracture	Caudal horn medial meniscus	2 stitches: 2 vertical	2 polypropylene	42	Breakage of one suture during insertion through meniscus	X-Porous TTA
5	Labrador	F	36	4 y 10 m	Left	CCL rupture	Caudal horn medial meniscus	3 stitches: 2 vertical, 1 horizontal	1 polypropylene 2 PDS	85	Difficult visualization Breakage of one suture during insertion through meniscus	X-Porous TTA
6	Mongrel	M	28	7 y 1 m	Right	CCL rupture	Caudal horn medial meniscus	2 stitches: 2 vertical	2 polypropylene	52	None	X-Porous TTA
7	Dobermann	F	32	1 y 10 m	Right	CCL rupture	Caudal horn medial meniscus	3 stitches: 2 crossed, 1 vertical	2 polypropylene 1 PDS	72	None	X-Porous TTA
8	Dobermann	F	32	5 y 11 m	Left	CCL rupture	Caudal horn medial meniscus	3 stitches: 3 vertical	1 polypropylene 2 PDS	83	None	X-Porous TTA
9	Boxer	M	35	10 y 2 m	Left	CCL rupture	Caudal horn medial meniscus	2 stitches: 2 vertical	2 polypropylene	45	None	X-Porous TTA
10	Cane Corso	M	48	1 y 5 m	Left	CCL rupture	Caudal horn medial meniscus	3 stitches: 3 vertical	1 polypropylene 2 PDS	68	None	X-Porous TTA

## Data Availability

The data generated in this study are available from the corresponding author upon request.

## Abbreviations

K-wire	Kirschner wire
TPLO	tibial plateau leveling osteotomy
TTA	tibial tuberosity advancement
XPTTA	X-Porous TTA
CCLR	cranial cruciate ligament rupture
M:	male
F	female
PDS	polydioxanone
